# Editorial: Nutritional modulation of central nervous system development, maintenance, plasticity, and recovery

**DOI:** 10.3389/fnins.2023.1332191

**Published:** 2023-11-17

**Authors:** Ilaria Bertocchi, Silvia Turroni, Fausto Chiazza

**Affiliations:** ^1^Department of Neuroscience Rita Levi Montalcini, University of Turin, Turin, Italy; ^2^Neuroscience Institute - Cavalieri-Ottolenghi Foundation (NICO), Laboratory of Neuropsychopharmacology, Turin, Italy; ^3^Unit of Microbiome Science and Biotechnology, Department of Pharmacy and Biotechnology, University of Bologna, Bologna, Italy; ^4^Department of Pharmaceutical Sciences, University of Eastern Piedmont, Novara, Italy

**Keywords:** nutrition, brain, gut-brain axis, cognition, mood

There is no doubt about the negative impact of an unhealthy diet on Central Nervous System (CNS) development and homeostasis (Cordner and Tamashiro, 2015; Chiazza et al., 2021). Indeed, increasing evidence links modern diets (high in saturated fat, refined carbohydrates and processed foods) with cognitive and memory deficits, increased incidence of mood disorders and risk of neuroinflammation and neurodegenerative diseases (Gauci et al., 2022; Oberto et al., 2022).

The aim of the Frontiers in Neuroscience Research Topic (RT) “*Nutritional modulation of central nervous system development, maintenance, plasticity, and recovery*” was to spotlight the latest advances and research outputs highlighting the important role of nutrition on the CNS, during development, maintenance processes, plasticity, and recovery. We provide an overview of this RT, including 2 original research articles, 1 brief research report and 1 review article.

In this RT, Slomp et al. used a state-of-the-art imaging approach in mice to investigate the response of GABAergic neurons (Vgat positive) to diet in a key region regulating feeding behavior, the lateral hypothalamus (LH). Their main aim was to understand whether this neuronal population might be affected by a free-choice high-fat diet (fcHFD). Their findings revealed that fcHFD reduced overall LH^Vgat^ neuronal activity, but did not disrupt the increase in activity induced by sucrose consumption. Previous studies have reported diet-induced changes in LH activity and, interestingly, the same research group observed that fcHFD altered the response of glutamatergic LH neurons to sucrose drinking (Koekkoek et al., 2021). The results now reported demonstrate that short-term consumption of saturated fat can also influence the activity of the GABAergic neuronal population in the LH. However, it remains unclear how the fatty diet can dampen GABAergic tone without interfering with the ability of LH^Vgat^ neurons to recognize and react differently to sugar solution and water consumption. GABAergic neurons within the LH are influenced by different external and internal stimuli, including specific responses to taste reactivity, palatability and dopaminergic and opioid transmission. Given the heterogeneity of the LH GABAergic neuronal population, the mechanisms involved may therefore be multiple, complicated, and interconnected; this supports the case for further research.

In the context of this RT, microbiota-mediated effects of diet on brain performance and CNS homeostasis (via the so-called “gut-brain axis”) have been ascribed a leading role. Indeed, the community of more than 10 trillion microorganisms residing in our gastrointestinal tract can influence whole-body health by converting dietary components into bioactive small molecules that can enter the bloodstream, be further metabolized by the host, modulate the immune system, and reach distal organs (Turroni et al., 2018).

Salami and Soheili reviewed the available literature on the association of gut microbiota and probiotic supplements with behavioral, electrophysiological, biochemical, and histological aspects of the hippocampus (known to be involved in cognition, emotion, and anxiety). The 139 experimental studies and clinical trials included in their systematic review confirmed the ability of gut microbiota (and probiotics) to influence synaptic activity in hippocampal neural circuits and related behaviors. They also summarized the main molecular mechanisms known to date, including the production and modulation of neurotrophins, neurotransmitters and receptors, the regulation of intracellular molecular processes, the balance of pro-inflammatory/anti-inflammatory and pro-oxidative/anti-oxidative factors, and the preservation of histological stability of the hippocampus. However, as the authors pointed out, this field of research is still in its infancy and will require intensive future efforts to make practical advances in microbiota-based diagnosis and/or treatment of brain disorders.

The importance of continuing research into the gut-brain axis has been picked up by Freijy et al., who participated in this RT by evaluating the efficacy of probiotic supplements (a multi-species formulation of bifidobacteria and lactobacilli) on mental health. This formulation was tested in a randomized controlled trial (“The Gut Feelings”) against a high-prebiotic diet and a synbiotic (as a combination of probiotics and prebiotics) in 119 adults with moderate psychological distress (and low prebiotic intake). After 8 weeks of treatment, the authors found a reduction in total mood disturbance (the primary outcome) and improvements in anxiety, stress and sleep (secondary outcomes) only in the prebiotic group. While larger trials in both clinical and non-clinical populations are needed, their findings strongly support the importance of a healthy diet (rich in whole plant foods) as a simple and cost-effective means of promoting mental health.

In addition to diet, healthy physical habits are known to delay brain downfall, especially during aging. However, physical activity is not always feasible for elderly people, particularly in frail conditions such as after ischemic events. Therefore, an active research area is investigating therapeutic targets and strategies for developing exercise mimetics, particularly in CNS disorders.

On this basis, Ragni et al. have investigated whether dietary supplementation with a balanced essential amino acid mixture (BCAAem) influenced mitochondrial biogenesis and antioxidant response in the hippocampus of middle-aged mice compared to those evoked by treadmill exercise training. Here they have shown that BCAAem induced eNOS expression, mitochondrial biogenesis markers, and antioxidant genes in the mouse hippocampus to a degree comparable to that induced by exercise training. Furthermore, this diet supplementation promoted mitochondrial biogenesis in mouse cortical neurons *in vitro* and protected them from ischemic insult through mTOR and eNOS-mediated mechanism(s). This last finding is particularly interesting as the risk of ischemic stroke onset is known to be strongly increased by a metabolic derangement, and many pharmacological strategies have been attempted in this regard (Chiazza et al., 2018). Thus, also in this field, future investigations using *in vivo* models of cerebral ischemia are needed to deepen the role of BCAA-based metabolic modulators in stroke prevention or as a valuable aid in multidisciplinary post-stroke rehabilitation in humans.

As highlighted by this RT, a better understanding of how diet may influence CNS homeostasis, including through gut-brain signaling, is pivotal to provide us with effective tools to address unhealthy dietary behaviors and associated brain disorders.

## Author contributions

IB: Writing—original draft, Writing—review & editing. ST: Writing—original draft, Writing—review & editing. FC: Writing—original draft, Writing—review & editing.
